# Cytotoxicity, Genotoxicity, and Phytotoxicity of Tannery Effluent Discharged into Palar River Basin, Tamil Nadu, India

**DOI:** 10.1155/2015/504360

**Published:** 2015-12-29

**Authors:** Suki Roy, Lubbnaz Nagarchi, Ishita Das, Jayasri Mangalam Achuthananthan, Suthindhiran Krishnamurthy

**Affiliations:** Marine Biotechnology and Bioproducts Lab, School of Biosciences and Technology, VIT University, Vellore 632014, India

## Abstract

Ambur, a town located on the banks of Palar River, is considered one of the most polluted areas in India and occupied by hundreds of tanneries and leather product units. The present study was designed to evaluate the toxic effect of discharged tannery effluent (TE) on model agricultural crops, ecofriendly microorganisms, and human blood cells. The phytotoxic effects of TE tested on* Allium cepa *and* Lemna minor *revealed inhibition of root growth and significant reduction in number of fronds, protein, and chlorophyll content. Moreover, TE induced chlorosis and tissue necrosis in* Nostoc muscorum* at low concentration (10%). TE has also negative impact on ecofriendly microorganisms,* Bacillus thuringiensis*,* Rhizobium etli*, and* Aspergillus terreus* which play an important role in the nutrition of plant growth. The genotoxicity of TE was investigated in human leukocytes which showed interference with normal mitotic division with subsequent cell lysis. It also intervened with the normal replication process and induced micronucleus formation in the healthy leukocyte. 5% concentration of TE has been revealed to be toxic to erythrocytes. From this study TE found in the Palar River of Ambur has adverse effects on all the three levels of organisms in ecosystem even at lower concentrations.

## 1. Background

Indiscriminate discharge of untreated waste water directly or indirectly into aquatic bodies may result in polluted water resources. This would adversely affect humans and other living systems. In some regions, the environment is under increasing pressure from solid and liquid wastes emanating from the leather industry. Tannery effluents are ranked as the highest pollutants among all industrial wastes [1]. India is the third largest producer of leather in the world having about 3000 tanneries with annual processing capacity of 0.7 million tonnes of hides and skin [2]. The metals generally present in tannery effluents (chromium, aluminium, zirconium, etc.) are all classified as having a high/moderately acute or chronic toxic effect on organic life [3]. These are inevitable by-products of the leather manufacturing process and cause significant pollution unless treated in some way prior to discharge. Moreover, the leather industry mainly causes high influx of chromium into the biosphere which contributes 40% of the total industrial use. Treated wastewater discharged from tanning industries contains high level of biochemical oxygen demand (BOD), chemical oxygen demand (COD), electrical conductivity, and heavy metals especially Cr above permissible levels making it potentially toxic [4].

The Palar River is one of the major rivers flowing through Vellore district (120 km in length with 4710 area of river basin). The Palar valley, one of the most desirable locations of tanning industry in India, is located near Ambur, India. There was a linear growth of tanning industry in Palar valley as well as the waste discharged to the river. The chemicals released by the leather tanneries directly make way into Palar River (perennial stream that is often completely dry) and traversing across Ambur has led to ground water pollution thereby disturbing the ecological balance [5].

Elevated chromium concentration in the effluents from tanneries poses a serious environmental concern in Vellore district. Depending on its oxidation state and concentrations, chromium can be either beneficial or toxic to animals, plants, and humans [6]. Cr (III) is considered an essential component of a balanced human and animal diet for preventing adverse effects in the metabolism of glucose and lipids [7]. High concentrations of tannery effluent can interfere with several metabolic processes because of its ability to coordinate various organic compounds resulting in inhibition of metalloenzyme systems [8]. The presence of heavy metals in tannery effluent causes toxic effect to living organisms as reported by Siyanbola et al. (2011) [9]. The toxic metals in tannery effluent cause lethal effects, genotoxicity, mutagenicity, and carcinogenicity to microorganisms, aquatic organisms, plants, animals, and human beings [10].

To identify the key toxic compounds and underlying toxic potential of the compounds present in tannery effluent, various toxicity assays should be performed. The present study was to evaluate the toxic effect of tannery effluent on microorganisms (*Bacillus, Rhizobium,* and* Aspergillus*), Cyanobacterium (*Nostoc muscorum*), plants (*Allium cepa*,* Lemna minor*), and red blood cells (RBC).

## 2. Materials and Methods

### 2.1. Study Area and Effluent Collection

The effluent sample was collected from highly contaminated tannery effluent discharge site in Palar River basin with geographical location of 12.78°N latitude 78.7°E longitude. Sample sites were well-known for their high pollution rate for the past 25 years and before that this basin was agricultural field. For the last 25 years, effluents from most of the leather industries are being discharged into the river, road sides, and agricultural fields. Effluent sample was collected in acid washed sterile plastic containers of 5-litre capacity from the TE accumulated site. The collected samples were transported to the laboratory aseptically and stored in sterile condition until further experiments were conducted.

### 2.2. Physicochemical Properties of Effluent

The collected sample was analyzed for a number of physicochemical parameters such as colour, pH, total dissolved solids (TDS), and total solids (TS) employing standard methods. Determination of BOD (biochemical oxygen demand) was done by Winkler's iodometric method [11] and COD (chemical oxygen demand) was determined after oxidation of organic matter in strong tetraoxosulphate VI acid medium by K_2_Cr_2_O_7_ at 148°C, with back titration (titrimetric method) [12]. The dissolved oxygen (DO) content was determined before and after incubation. The chromium, zinc, nickel, lead, and magnesium content were estimated by atomic absorption spectrophotometer and identified by limit test (http://www.epa.gov/). Sodium and potassium content was measured by flame photometric method [13]. PO_4_, NO_2_, NO_3_, and chlorine were determined by ion chromatography [14].

### 2.3. Microbiological Activity of Tannery Effluent

The minimum inhibitory concentration (MIC) of TE for each microorganism (*Bacillus thuringiensis*,* Rhizobium etli*, and* Aspergillus terreus*) was determined. Pure culture* Bacillus thuringiensis* was cultured in Mueller-Hinton broth,* Rhizobium etli* in PY rich medium [15, 16], and* Aspergillus terreus* in potato dextrose broth medium. The test microorganisms were obtained from Marine Biotechnology and Bioproducts Lab, VIT University, Vellore, and were cultured overnight in respective media prior to the experiment. The zone of inhibition of tannery effluent was determined by Kirby-Bauer well diffusion method [17]. The test microorganisms were plated and different concentrations of tannery effluent were added to the wells. The zone of inhibition was observed after 48 hours of incubation. To detect the MIC the collected TE was diluted with respective media and 100 *μ*L of the TE of various concentrations (0–100%) was added to each well of 96-well microplate and test organisms were inoculated. This was allowed for incubation at 28°C for 24–72 hrs and optical density (OD) was measured at 595 nm. The lowest concentration of tannery effluent that inhibits the growth of microorganisms was considered the minimum inhibitory concentration (MIC) of TE against the organism tested [18]. Distilled water was used as control and the experiments were done in triplicates.

### 2.4. Toxicity Assessment Using Cyanobacteria

*Nostoc muscorum* was used for cyanobacterial bioassay.* Nostoc muscorum* was grown in flask containing 200 mL of nitrogen-free modified Chu-10 medium [19]. Culture was aerated 2-3 times per days. The cells in log phase (4–6-day-old culture) were added to four different dilutions of TE (0, 25, 50, and 100%). Samples were incubated and cells were harvested after 7-8 days by centrifugation at 10000 rpm for 30 min at 4°C [20]. Samples were examined for different growth parameters, such as total chlorophyll content and protein content by spectrophotometric and fluorometric method. Protein content was analyzed as described earlier [21]. The experiments were performed in triplicates.

### 2.5. Cultivation of* Allium cepa*

Healthy onion bulbs of* Allium cepa* were purchased from local supermarket and washed in running water to remove contamination on outer covering. After drying onion bulbs (1.5 to 2.2 cm in diameter) were rinsed thoroughly with tap water and the outer scales were removed. Old root remnants were removed without disturbing the root primordia. Initially the onions were grown in distilled water for 24 hrs and then the actively dividing root tip cells of onion were exposed to different TE concentrations (5, 10, 15, 20, 25, 30, 35, 40, 45, 50, and 55%) along with a control (distilled water). Each concentration was set up in three replicates. Every 24 hours the test solutions are replaced by fresh ones. The plants were harvested after 48 hr to evaluate the phytotoxic effects. The root length of* Allium cepa*, grown at different concentrations of TE, was measured with the help of a meter scale and mean root length was calculated. Minimum root inhibition was calculated as EC_50_.

### 2.6. Cultivation of* Lemna minor*

Fresh duck weeds (*Lemna minor* L.) were collected from Department of Horticulture at VIT University and the assay was performed at room temperature. The fronds were separated carefully and disinfected with 1% of sodium hypochlorite solution. About 2 grams of fronds was then inoculated in Quarter Coic and Lessaint solution [22, 23] along with TE (5%, 10%, 15%, and 25%) for 96 hrs. The medium without TE served as control.

### 2.7. Protein Extraction

Total soluble proteins were determined according to Lowry's method. This is done by collecting the TE treated fronds from each test concentration and by grinding using pestle and mortar in 5 mL of potassium phosphate buffer. The tubes were centrifuged at 12,000 rpm for 20 min. The clear supernatant was collected and the total protein content was estimated by Lowry's method [21].

### 2.8. Determination of Photosynthetic Pigments and Soluble Protein

Approximately 150 mg of* Lemna* fronds treated with different TE concentrations were homogenized on ice in 3 mL of 66 mM phosphate buffer (pH 7.2) by adding 10 mM KCl sequentially. The homogenate was extracted with 80% cold acetone and stored for standardization. Chlorophyll content was determined by the method of Arnon (1949) [24]. The absorbance of pigment extract was measured at wavelength of 470, 537, 647, 663, and 730 nm using UV-Vis spectrophotometer. The contents of chlorophyll a, chlorophyll b, and carotenoid were calculated in accordance with experimental equations as described by Arnon (1949) [24].

### 2.9. Genotoxicity Analysis Using Leucocytes

#### 2.9.1. Determination of Chromosomal Abnormality

Heparinized peripheral blood obtained from a healthy non-smoking donor was used for all the experiments. About 5 mL of venous blood sample from healthy donor (O +ve) was collected. Five vials each containing 0.5 mL of blood were inoculated in 5 mL of RPMI 1640 medium (Himedia, India) supplemented with 20% of fetal bovine serum, 100 IU/mL penicillin, 100 *μ*g/mL streptomycin, 0.5 mg/mL L-glutamine, and 6 *μ*g/mL of phytohemagglutinin (PHA) which were added under aseptic condition. The culture vials were incubated at 37°C for 72 hours. The cultures were shaken every 24th hour and carbon dioxide was released once in 24 hours. At the 48th hour of incubation, the cultures were treated with TE (5%, 10%, 15%, and 20%) and incubated for the next 24 hours at 37°C. A positive control with mitomycin-C and a control without tannery effluent were maintained. After 70 hrs the dividing cells were arrested at metaphase by adding 2 drops of 0.1% colchicine (Sigma, India) and incubated further for 2 hrs. After 72 hrs of incubation the cells were centrifuged, the supernatant was gently discarded, and 6 mL of prewarmed hypotonic solution (KCl 0.075 M) was added to swell the cells. After incubation, the contents were centrifuged at 2000 rpm for five minutes and supernatant was discarded, treated twice with Carnoy's fixative (3 : 1 ratio of methanol : acetic acid), and mixed vigorously. After fixation all the contents were centrifuged and supernatant was discarded and washing step was continued till the pellet turned white. The slides were prepared and kept in incubator at 37°C for drying. Later, the slides were stained with 10% Giemsa stain and destained with deionised water [25–27].

#### 2.9.2. Cell Counting

The cells were counted in every vial using haemocytometer by staining the cells with Trypan blue (Himedia, India). The estimations were made by scoring the cells in duplicates for each concentration.

#### 2.9.3. Micronuclei Detection

For micronuclei detection, 0.5 mL of blood was added to 5 mL of RPMI-1640 medium supplemented with 15% of fetal bovine serum (FBS). The five vials of lymphocyte culture were incubated for 72 hours at 37°C. The cultures were shaken every 24th hour and carbon dioxide was released. At the 24th hour of the experiment different concentrations (5%, 10%, 15%, and 20%) of tannery effluent were added and a control (without tannery effluent) was also maintained. For micronucleus preparation cytochalasin B (Sigma, India) was added to each vial at a final concentration of 4 *μ*g/mL after 44 hours after culture initiation [28–30]. The vials were kept for incubation for another 24 hrs at 37°C and subjected to a brief cold hypotonic (0.056%) KCl treatment. Micronucleus slides were made by fixation in methanol : acetic acid (3 : 1) and the slides were further stained using 2% alkaline Giemsa. The cells were observed under light microscope and binucleated cells were scored for each.

#### 2.9.4. *In Vitro* Hemolytic Assay

*In vitro* haemolytic activity was analyzed as described by Suthindhiran and Kannabiran (2009) [31]. Human erythrocytes were prepared from the peripheral blood (A+) of a healthy volunteer. About 5 mL of blood was collected in a heparinized tube and 2% erythrocyte suspension was prepared by washing the cells three times with 20 mM Tris-HCl containing 25 mM NaCl (pH 7.4). The cells were further washed three times in nine volumes of sterile 0.9% saline solution. After each washing, cells were centrifuged at 2000 rpm for 5 min and the supernatant was discarded. The final pellet was diluted to 1/9 (v/v) in sterile 0.9% NaCl saline solution then to 1/24 (v/v) in sterile Dulbecco's phosphate buffer saline (pH 7.0) containing 0.5 mM boric acid and 1 mM calcium chloride [32]. The test was carried out in 96-well plate. To each well 100 *μ*L of 0.85% sodium chloride solution containing 10 mM calcium chloride was added. To this 100 *μ*L of tannery effluent in different concentrations (5%, 10%, 15%, and 20%) was added. 100 *μ*L of 2% erythrocyte suspension was added to each well and incubated at room temperature for 30 min. The contents were then centrifuged and absorbance of the supernatant was measured at 540 nm against a reagent blank in which the effluent was substituted by saline. 20 mL of 0.1% Triton X-100 in 0.85% saline served as positive control and negative control contained distilled water. The percentage of hemolysis was calculated [33] and the average value was calculated from triplicate assay.

## 3. Results

### 3.1. Physicochemical Characteristics of Tannery Effluent

The colour of the effluent appeared as dark brown with pH of 7.6 (Table 1). The biological oxygen demand (BOD) and the chemical oxygen demand (COD) of the effluent were found to be 526 mg/L  and 2986 mg/L, respectively. Dissolved oxygen values of the effluent were measured as 36.21 mg/L. The effluent had high total dissolved solids (TDS) which was about 6395 mg/L. The data represented in Table 2 showed higher values of metals and anions in the effluent than the recommended values. The effluent was found to have lead, iron, copper, zinc, manganese, and arsenic concentrations much higher than regulatory limits set by the US Environmental Protection Agency [34]. The concentration of chromium and nickel in the effluent was at highest levels. In general, the trend of metal availability in the effluent sample was Ni > Cr > Zn > Fe > Mn > Mg > Co > Cu > Pb > Cd > Ar. The tannery effluent also had higher load of sulphate and chloride of around 776 mg/L  and 187 mg/L, respectively.

### 3.2. Effect of Tannery Effluent on Microorganisms

Growth of all the tested microorganism (*Bacillus thuringiensis*,* Rhizobium etli*, and* Aspergillus terreus*) were inhibited by TE. The inhibition of growth was observed in bacteria and fungi at a concentration of 30%. The results showed a concentration dependent growth inhibition and zone of inhibition of each organism was determined as shown in Figure 3. The cyanobacterial bioassay indicated that, with increase in concentration of TE, chlorophyll content of the* Nostoc muscorum* significantly decreased as shown in Table 5. Among all parameters chlorophyll was found to be the most sensitive as it showed chlorosis followed by tissue necrosis even at low concentration (10%) of TE.

### 3.3. Effect of Tannery Effluent on* Allium cepa* and* Lemna minor*

The root growth inhibition in* Allium cepa* has been observed at different concentrations of effluent (Table 3). The root growth of the* Alium cepa* is inhibited by TE even at lower concentrations. The reduction in root length implies inhibition of root growth. About 47.3% reduction in root growth was observed at lower concentration of TE (5%). The percentage root growth was found to decrease (52.7, 47.2, 43.6, 38.1, 27.2, 16.3, 9.1, and 3.6%) when the concentration of TE increased (5, 10, 15, 20, 25, 30, 35, and 40). Treatment with TE showed significant inhibition of root growth and no root growth was observed in* Allium cepa* treated with tannery effluent concentration beyond 40% (Table 3). The EC_50_ (the concentration of effluent causing 50% of total damage) of* Allium cepa* treated with tannery effluent was found to be 7.79%.

Different concentrations of tannery effluent affected the number of fronds in* Lemna minor* (Table 4). The control showed an increase in the number of fronds after 96 hours of exposure to tannery effluents but all the tannery effluent treated* Lemna minor* showed significant decrease in the number of fronds in same time period. The number of fronds of* Lemna minor* treated with lowest concentration of TE (5%) reduced from 42 to 38 (9% reduction) whereas the exposure of highest concentration of TE (20%) caused a reduction of 57% after 96 hours. The control showed a 32% increase in number of fronds. Some of the effluent treated* Lemna minor* found to have lost its natural green colour and turned white which indicated the death of fronds and chlorosis.

The protein content and photosynthetic pigments of* Lemna minor* were affected by tannery effluent (Table 5). The protein content reduced to 52 *μ*g/mL, 50 *μ*g/mL, 38 *μ*g/mL, and 12 *μ*g/mL with tannery effluent concentration of 5%, 10%, 15%, and 20%, respectively. The reduction in protein content was more at the highest concentration of tannery effluent (20%). The chlorophyll a and carotenoid content also declined considerably in all concentrations of tannery effluent except the lower concentration (5%), which showed only a slight decrease. The chlorophyll a content was 86% retained after treating with 5% TE but only 9.5% of chlorophyll a was present after exposing to 20% TE. The carotenoid concentration of control was estimated to be 3.474 *μ*g/mL. The pigment content showed 15% decrease at the lowest TE concentration (5%). The increase in TE concentration continued to decrease the carotenoid content in which the highest concentration of TE (20%) showed 90% decrease in carotenoid. However, the chlorophyll b content decreased only in higher concentration of tannery effluent. The highest concentration of TE (20%) showed a 36% decline in chlorophyll b content whereas exposure with lower concentrations of TE caused only slight decrease.

### 3.4. Effect of Tannery Effluent on Human Blood Cells

#### 3.4.1. Cell Viability

The number of viable cells was found to be 6.25 × 10^6^ in the control. Progressive decrease in cell number from 5.5 × 10^6^ (5% TE) to 1.8 × 10^5^ (20% TE) was observed with increased exposure to TE. Significant decrease in cell count was found at 15% and 20% of TE treated with culture (Table 6). The cell population at these volumes was found be to almost half of the control cell population.

#### 3.4.2. Chromosomal Aberrations

**Figure 1:**
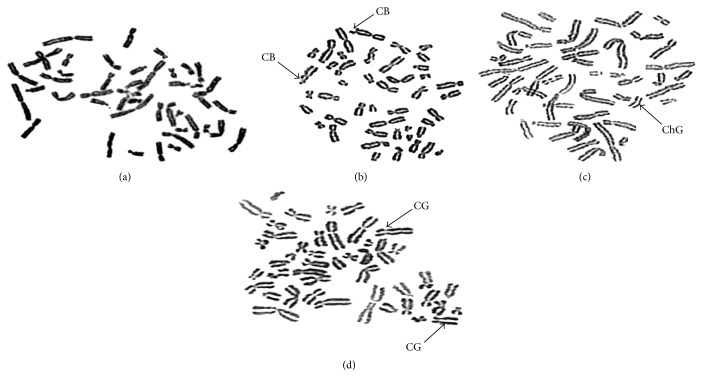
Chromosomal aberrations in human blood cells due to tannery effluent exposure: (a) Control; (b) human blood cells treated with 20% TE; arrow indicates chromatid breaks (CB); (c) human blood cells treated with 20% TE; arrow indicates chromatid gaps (ChG); (d) human blood cells treated with 20% TE; arrow indicates chromosomal gaps (CG).

Normal chromosomes were observed in control. However, in chromosomes of TE treated cells, aberrations were observed. Among 50 metaphases counted at least one deletion was observed in one of the chromosomes which were treated with 20% of TE. At least three types of structural aberrations, namely, chromosome breaks (Figure 1(b)), chromosomal gaps (Figure 1(c)), and chromatid gaps (Figure 1(d)), were observed in cells treated with 20% TE. Fragmentation of the chromosomes in cultures with 10%, 15%, and 20% was also observed.

#### 3.4.3. Micronuclei Detection

Micronuclei were found in the cells obtained from the cultures containing 15% and 20% of TE. Micronucleus was found adjacent to the nucleus but was smaller in size than the nucleus. In control the micronucleus was found to be absent. Presence of the micronucleus is an indication of cellular damage; hence, TE is found to induce toxicity to human leukocyte and induces damage in the cells at a concentration of 15% and 20%, respectively (Table 6).

**Figure 2:**
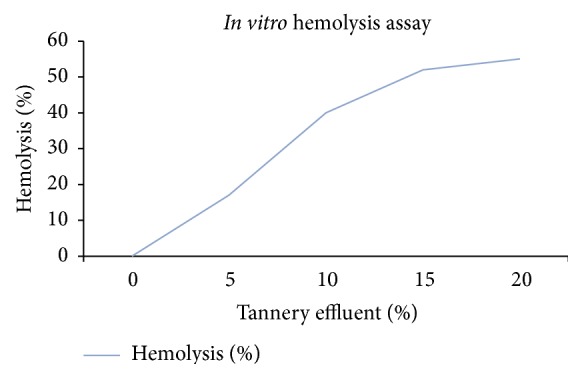
Percentage of hemolysis in* In vitro* Hemolytic Assay indicating the concentration of tannery effluent that can cause 50% of hemolysis.

**Figure 3:**
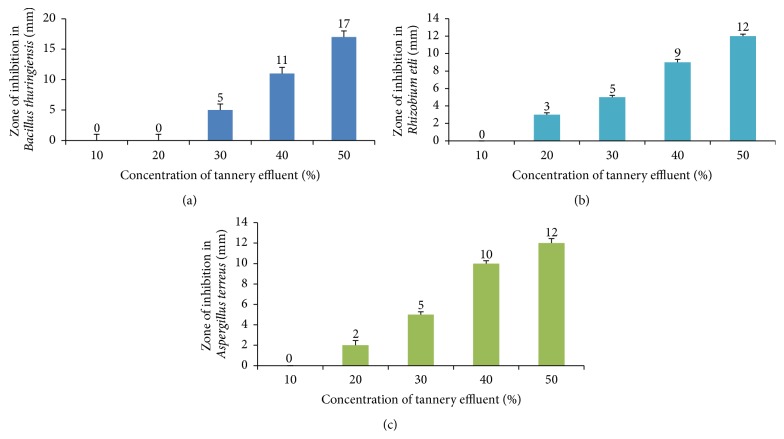
Effect of tannery effluent on ecofriendly microorganisms. (a) Zone of inhibition in* Bacillus thuringiensis* (mm). (b) Zone of inhibition in* Rhizobium etli* (mm). (c) Zone of inhibition in* Aspergillus terreus* (mm).

*In Vitro Hemolytic Assay*. Hemolysis was observed in cultures treated with TE and was found to be dose dependent. Moderate hemolysis was found at 5% of TE but significant decrease in erythrocyte population was observed at 10%. The positive control showed 94% of hemolysis as compared to the tannery effluent. Furthermore, considerable amount of hemolysis was observed at 15% of TE in which the effluent present in the culture induced cell death of approximately 50% of erythrocyte population (Figure 2). Hence, 20% and above of tannery effluent was found to be highly toxic to human erythrocyte.

## 4. Discussion

Overall data emphasize on the inhibitory and toxic effect of TE on plants, human blood cells, and microorganisms. The biological oxygen demand (BOD) values of the effluent were higher than the recommended acceptance level set by Federal Environmental Protection Agency (FEPA) [35] for the discharged effluents of tanneries and textile into water bodies (50 mg/L). The COD of the effluent was also found to be higher than United States Environmental Protection Agency (USEPA) standard (1000 mg/L) for discharge of TE into surface water [34]. These high levels of BOD and COD values observed in the effluent were due to high quantity of organic matter from a variety of chemicals used in the soaking, tanning, and posttanning processing of leather. It has been reported that only about 20% of the chemicals used in the tanning method is absorbed by leather and the rest is unconfined as waste thereby raising the levels of BOD in the effluent [36]. It should be noted that high BOD levels in tannery effluent could even cause rapid depletion of dissolved oxygen if directly discharged into surface water. Dissolved oxygen values were relatively less in the analyzed sample due to the increase of organic matter. Total dissolved solids (TDS) were higher than standard value recommended by USEPA [33]. High TDS detected could be credited to the strong colour (from the a range of dying stuff being used in the tannery mills) and they might be the main sources of the heavy metals as higher heavy metals concentrations in river sediments could raise suspended solids concentrations [37]. Any increase in free ion concentrations in the outside surroundings as well as concentrations of other environmental stressors would lead to osmoregulation imbalances in aquatic animals such as fishes. In our study high concentration of heavy metals was observed in the TE. All the values of metals and anions in the effluent were higher than USEPA standard which might be due to the chemicals used by tanneries for chrome tanning process [34]. The concentration of other anions also was found to be higher than USEPA standard values [34]. The high levels of nitrate and nitrite in the TE might be due to the chemicals used in the tanning process which contains nitrogen as element and the nitrogen content in proteinaceous substance [38]. The high level of sulphate was observed in the effluent because of the use of sulphuric acid or product with high sodium sulphate content during tanning process [39].

Higher concentration of TE leads to phytogenic toxicity in tested plants. The main symptoms of the toxicity include reduction in root length, leaf chlorosis, inhibition of seed germination, and depressed biomass [4]. The effect of collected TE on* Allium cepa* showed a significant reduction in root length, decrease in dry weight, increase in root diameter, and increase in root hairs. The estimated EC_50_ value of* Allium cepa* exposed to TE was found to be 7.79% which is almost similar to the findings of Gupta et al. (2011) [40]. The inhibition of root growth may be due to suppression of root cell division/root elongation or the extension of cell cycle in the roots [41]. It was also observed that the TE treatment resulted in a significant decline of frond number, chlorophyll, and protein content in* Lemna minor.* Major toxicity of TE in plant can be observed with respect to photosynthetic pigment, photosynthesis, and protein content [42]. In our study, the TE treatment showed a significant decrease in protein and pigment contents. The protein and pigment content of* Lemna minor* gradually decreased with an increasing concentration of TE. It was already reported that the presence of heavy metals results in chlorosis, necrosis, and change in the concentration of essential minerals in plants [43, 44] and in cyanobacteria [45]. Reduction in photosynthesis may result from the stomatal closure and alteration in the ultrastructure of chloroplast. High concentration of cobalt, chromium, and copper greatly affect the concentration of iron, chlorophyll “a” and chlorophyll “b”, protein, and catalase activity in plants [23]. Chromium is known as the toxic, mutagenic, carcinogenic, and teratogenic component of tannery effluent and it is a strong oxidizing agent that causes severe damage to cell membranes [46]. Chromium toxicity can reduce the size of the epidermal layer of the antenna complex in chloroplast which can ultimately decrease the chlorophyll a content [44]. It can also lead to impairment and denaturation of the proteins of the outermost part of antenna reducing the chlorophyll b content of the plant. Under the chromium stress, there can be inactivation of enzymes which are involved in the biosynthesis pathway of chlorophyll, ultimately reducing the chlorophyll content as a whole [47].

The TE showed toxicity to the tested cyanobacteria* Nostoc muscorum* by reducing the total chlorophyll content. The toxicity might be due to the presence of high level of chromium in TE. Cr (VI) reduces the quantum yield of PSII reaction centre and thus affects the chlorophyll content. High chromium content can also lead to DNA damage and may lead to the death of algal cells. An increase in TE concentration caused a significant decrease in the cell density and cell number. The exposure to tannery effluent in plants affects their metabolic process in several ways including reduction in growth, photosynthesis, chlorophyll content, and degradation of chloroplast and mitochondria.

The tannery effluent was found to be toxic to human leukocyte. It hinders the cytokinesis of the cells and mitotic index is expected to reduce considerably. In this study, even the lower concentrations of TE induced the potential toxicity to healthy human leukocytes. Cell counts of the leukocyte were reduced to almost 50% at 20% TE (Table 6). The TE interferes with the normal mitotic division, inducing cell death. In addition, tannery effluent was also found to induce chromosomal aberrations at all the tested concentrations. These results can be explained by the condition that TE might also interfere with the normal replication process and may also induce mutations that could be temporary or permanent [48]. This study focused further on effect of tannery in inducing micronucleus formation in the healthy leukocyte. Micronucleus was observed in cells exposed to 10% and 20% of TE. Since the presence of micronucleus indicates cellular damage, the TE can be considered toxic to the healthy human leukocyte even at small concentrations. The tannery effluent was also found to be toxic to red blood cells (erythrocytes) at the minimum concentration of 5%. The hemolysis was found to be dose dependent and cause 50% cell death at 10% TE. This result may be explained on the basis of the studied toxicity of chromium to the cells that forms the main constituent of tannery effluent. This proves that exposure of RBCs to even small concentration of TE can lead to lethal effects [49].

Our study revealed that the TE was highly toxic to the tested microorganism (*Bacillus thuringiensis*,* Rhizobium etli*, and* Aspergillus terreus*). Growth of all the organisms was inhibited by TE even at lower concentrations (5%). The toxicity may be due to the heavy metals present in the tannery effluent inhibiting the growth and activity of the natural bacterial populations. It may be due the complex-formation of heavy metals with organic compounds [50, 51] and heavy metals accumulation [52].

## 5. Conclusions

From the result obtained, it can be concluded that the physicochemical parameters of the TE, namely, dissolved oxygen and total dissolved solids, COD, BOD, nickel, magnesium, chloride, potassium, nitrite, sulphur, and chromium, were found to be much above the permissible limits prescribed by USEPA. The discharged TE into the Palar River, Ambur, is highly toxic to microorganisms, plants, and human blood cells, as it drastically affected the growth of microorganisms, root, protein, and chlorophyll content of the plants and chromosomes of human blood cells. Therefore, it is suggested that the release of effluent from tannery industries should be done after proper treatment and recycled to the maximum possible level and disposal into water resources should be minimised. Since the experimental conditions were carried out using diluted form of effluent, it can be interpreted that the crude effluent is highly toxic. It can also act as major water and soil pollutant in the respective area which may drastically affect ecofriendly microorganisms, plants, and also human beings.

## Figures and Tables

**Table 1:** Physicochemical characteristics of the tannery effluent collected from Palar River (all values are mean range for three observations each).

Sr. number	Parameter	Tannery effluent (values)
1	Colour	Dark brown
2	pH	7.6
3	BOD	526 mg/L
4	COD	2985 mg/L
5	DO	36.21 mg/L
6	TDS	6395 mg/L

**Table 2:** Average concentration of metals and anions in the tannery effluent collected from Palar River (all values are mean range for three observations each).

Sr. number	Metals	Concentration in tannery effluent (mg/L)	USEPA guidelines (mg/L)
1	Lead	2.98	0.2
2	Iron	5.21	0.3
3	Cobalt	3.83	0.03
4	Copper	3.12	1.0
5	Zinc	5.73	1.0
6	Manganese	4.11	1.0
7	Chromium	9.21	0.1
8	Nickel	11.79	0.1
9	Arsenic	1.46	0.05
10	Magnesium	3.87	1.0
11	Cadmium	2.26	0.1
12	Nitrate	78	50
13	Nitrite	129	12
14	Phosphate	16.9	5
15	Sulphate	776	250
16	Chloride	187	200

**Table 3:** Effect of tannery effluent on root growth in *Allium cepa* (all values are mean ± SD range for three observations each). The EC_50_ was found to be 7.79%.

Concentration of tannery effluent (%)	Root length (cm)	Root growth (%)
0	5.5 ± 0.33	100
5	2.9 ± 0.27	52.7
10	2.6 ± 0.21	47.2
15	2.4 ± 0.29	43.6
20	2.1 ± 0.27	38.1
25	1.5 ± 0.21	27.2
30	0.9 ± 0.28	16.3
35	0.5 ± 0.31	9.1
40	0.2 ± 0.27	3.6
45	0	0
50	0	0
55	0	0

**Table 4:** Effect of tannery effluent on number of fronds in *Lemna minor* (all values are mean range for three observations each).

Concentration of tannery effluent (%)	Number of fronds	
	0 hour	96 hour
0	42	56
5	42	38
10	42	33
15	42	22
20	42	18

**Table 5:** Effect of tannery effluent on protein content and photosynthetic pigments of *Lemna minor *(all values are mean ± SD range for three observations each).

Concentration of tannery effluent (%)	Protein concentration (*µ*g/mL)	Chlorophyll a concentration (*µ*g/mL)	Chlorophyll b concentration (*µ*g/mL)	Carotenoids concentration (*µ*g/mL)
0	60 ± 0.30	6.687 ± 0.14	1.00 ± 0.40	3.474 ± 0.22
5	52 ± 0.26	5.79 ± 0.17	0.916 ± 0.30	2.95 ± 0.27
10	50 ± 0.28	0.907 ± 0.15	0.811 ± 0.10	1.946 ± 0.24
15	38 ± 0.29	0.738 ± 0.16	0.800 ± 0.22	1.044 ± 0.26
20	12 ± 0.27	0.636 ± 0.18	0.640 ± 0.20	0.360 ± 0.25

**Table 6:** Effect of tannery effluent on leukocytecell viability.

Volume of tannery effluent in culture (%)	Number of cells viable after treatment (cells/mL)
Control	1.25 ± 0.01 × 10^6^
5	1.1 ± 0.05 × 10^6^
10	8 ± 1.15 × 10^5^
15	7.7 ± 0.67 × 10^4^
20	3.7 ± 0.29 × 10^4^
